# Impact of COVID-19-related methadone regulatory flexibilities: views of state opioid treatment authorities and program staff

**DOI:** 10.1186/s13722-023-00417-7

**Published:** 2023-10-17

**Authors:** Shannon Gwin Mitchell, Julia Jester, Jan Gryczynski, Melanie Whitter, Douglas Fuller, Caroline Halsted, Robert P. Schwartz

**Affiliations:** 1 Friends Research Institute, Inc., 1040 Park Avenue, Baltimore, MD 21201 USA; 2 Mid Shore Pro Bono, Easton, MD USA; 3 National Association of State Alcohol and Drug Abuse Directors, Inc., Washington, D.C. USA

**Keywords:** Methadone treatment, Opioid treatment programs, Regulations, COVID, Opioid use disorder treatment

## Abstract

**Background:**

During the COVID-19 pandemic, federal regulations in the USA for methadone treatment of opioid use disorder (OUD) were temporarily revised to reduce clinic crowding and promote access to treatment.

**Methods:**

As part of a study seeking to implement interim methadone without routine counseling to hasten treatment access in Opioid Treatment Programs with admission delays, semi-structured qualitative interviews were conducted via Zoom with participating staff (N = 11) in six OTPs and their State Opioid Treatment Authorities (SOTAs; N = 5) responsible for overseeing the OTPs’ federal regulatory compliance. Participants discussed their views on the response of OTPs in their states to the pandemic and the impact of the COVID-related regulatory flexibilities on staff, established patients, and new program applicants. Interviews were audio recorded, transcribed, and a content analysis was conducted using ATLAS.ti.

**Results:**

All SOTAs requested the blanket take-home exemption and supported the use of telehealth for counseling. Participants noted that these changes were more beneficial for established patients than program applicants. Established patients were able to obtain a greater number of take-homes and attend individual counseling remotely. Patients with limited resources had greater difficulty or were unable to access remote counseling*.* The convenience of intake through telehealth did not extend to new program applicants because the admission physical exam requirement was not waived.

**Conclusions:**

The experienced reflections of SOTAs and OTP providers on methadone practice changes during the COVID-19 pandemic offer insights on SAMHSA’s proposed revisions to its OTP regulations.

*Trial registration* Clinicaltrials.gov # NCT04188977.

## Background

Methadone treatment provided through opioid treatment programs (OTPs) is the most highly regulated substance use disorder (SUD) treatment in the USA [15]. OTPs are overseen by the federal Substance Abuse Mental Health Services Administration (SAMHSA) and the Drug Enforcement Agency, as well as Single State Agencies for alcohol and drugs, state licensing agencies, and in some cases, county and local government agencies. Each state has a designated State Opioid Treatment Authority (SOTA) who is responsible for OTP oversight and federal regulation compliance within their state (National Association of State Alcohol and Drug Abuse Disorders, [25]).

Prior to the 2020 COVID-19 Public Health Emergency, OTP regulations contained certain requirements for treatment, including limitations on take home doses based on patient’s tenure in treatment and eight measures of progress (e.g., absence of “abuse” of drugs and alcohol, regular clinic attendance, lack of behavioral problems at the program), limitations on the clinical role of non-physician practitioners (e.g., nurse practitioners, physician assistants), restrictions on which disciplines can administer/dispense methadone, and requiring an in-person physical exam for admission. The regulations also required OTPs to provide an adequate availability of counseling, typically in-person. For some patients, these requirements for medication administration and counseling created barriers to treatment entry and retention [2, 40]. Overcoming these barriers is of considerable importance because methadone treatment is associated with reduced risk of illicit opioid overdose death [8, 15, 30, 36].

The COVID-19 pandemic fundamentally changed the way methadone treatment was delivered by limiting the number of patients in OTPs at any given time to allow physical distancing to reduce the possibility of transmission of the virus [4, 11]. In March 2020, in response to the onset of the pandemic in the USA, SAMHSA issued an emergency exemption to the OTP regulations which allowed states to request blanket permission for stable OTP patients to receive 28 days of take-home doses and up to 14 days of take-home doses for patients who were less stable but thought to be able to safely handle the self-administration of methadone [31, 32]. That exemption left the operational definition of “stable or “less stable” to the discretion of the OTP medical staff (SAMHSA, [31]). In addition, telehealth counseling, rather than in-person, was allowed [4, 6, 10, 11, 35]. Although these changes required state approval and implementation by individual OTPs, they reportedly demonstrated benefits to patients by ensuring on-going access to services during the pandemic without increasing risk of overdose among patients or in their respective communities [3, 6, 10, 16, 38]. In terms of admission requirements, the exemption permitted patients starting buprenorphine but not methadone treatment at OTPs to forgo the in-person physical exam. The rationale for not including methadone treatment admissions in this exemption was because methadone can cause more sedation that buprenorphine and therefore it was considered useful to have an in-person patient examination to detect any subtle evidence of somnolence.

### Parent study

The parent study was a NIDA-funded multi-site trial focused on improving accessibility to methadone treatment in six opioid treatment programs (OTPs) in four states in the USA. It examined the use of an implementation facilitation intervention (Ritchie et al. [26]) to reduce waiting times for admission by encouraging the use of interim methadone treatment, which does not require routine counseling, and other changes to their admissions process [18, 19]. The study included planned qualitative interviews of OTP staff and SOTAs who were directly involved in the implementation intervention. Interviews were focused on participants’ views of treatment access, the OTP admission process, and interim methadone treatment.

### Purpose of the present study

The Implementation Phase of the parent study began just prior to the mid-March 2020 COVID lockdown. The subsequent federal COVID-19 Public Health Emergency declaration permitted SAMHSA to allow states to request a blanket exemption to the federal OTP take home regulations in order to support physical distancing at the programs. To that end, SAMHSA also continued to allow the use of telehealth for counseling. The aim of the present study was to examine the views of OTP staff and SOTAs that emerged during the parent study’s qualitative interviews on the impact of the relaxation of OTP regulations and shift to telehealth on OTP staff, established patients, and people seeking admission.

## Methods

### Parent study overview

The parent study employed a modified stepped wedge design which included an Implementation Facilitation Phase. The first cluster of three OTPs were randomly assigned at the end of February 2020 to begin the implementation facilitation intervention to improve accessibility to treatment in programs unable to admit patients within 14 days. The second cluster of three OTPs began the intervention at the end of November and beginning of December 2020. All OTPs finished the intervention by June 2021. The entire Implementation Phase occurred during the federal COVID-19 Public Health Emergency declaration, which began on March 20, 2020.

The intervention was focused on implementing interim methadone treatment and other changes to admission procedures. Interim methadone treatment, methadone without routine counseling, is permitted under the extant OTP regulations for OTPs with admission delays due to inadequate amount of counseling staff. Randomized trials have shown it to be an effective alternative to waiting lists [28, 39]. The implementation facilitation intervention consisted of an External Facilitator (RPS) conducting all OTP site visits and providing academic detailing (non-commercial educational activities) while working with Local Champions appointed by the OTP Director. The investigators led separate remote Learning Collaboratives for each OTP cluster and their associated SOTAs.

The External Facilitator invited all the SOTAs and OTP staff who were directly involved in the implementation intervention to participate in one individual qualitative interview. He emailed them a copy of the IRB-approved information sheet for the interviews and invited them to schedule an appointment with an experienced interviewer (SGM). All agreed to participate. During appointments, the interviewer reviewed the information sheet with staff and obtained verbal informed consent prior to conducting the interview via Zoom. Participants were not financially compensated for completing the interview. Human Subjects oversight was provided by the Western Institutional Review Board (WIRB).

### Present study

#### Participants

SGM, an experienced qualitative researcher, conducted in-depth, semi-structured interviews with SOTAs from the 4 states, including one of the SOTA’s associates (N = 5) and OTP staff (N = 11) who were directly involved in the intervention. All interviews were completed at the end of the participating clinics’ implementation period in the parent study, with eight interviews conducted in the fall of 2020 and the final eight occurring in the summer of 2021. Leadership (OTP directors & Clinical Directors; n = 8) and Intake Staff (N = 3) from each of the participating OTPs in the parent trial were interviewed along with the SOTAs from the four participating states, for a total sample of 16 participants. The sample was entirely White, predominantly non-Hispanic (15/16) and female (n = 10/16).

#### Interviews

Interviews were conducted individually in all but two cases in which participants wished to be interviewed together to provide multiple perspectives. Interviews averaged 60 min in length and followed a semi-structured interview guide to examine the process of preparing to implement interim methadone, focusing on its feasibility and acceptability. However, since implementation activities coincided with COVID-19, many of the questions were impacted by changes occurring at the federal, state, community, and OTP levels to reduce the population’s exposure to the virus. These questions focused on aspects of care, such as staffing, treatment access for new and established patients, and methadone regulations. The study’s semi-structured interview guide accommodated the influence of these larger regulatory modifications by the addition of probes specifically querying for the impact of COVID-19-related changes.

#### Analysis

The interviews were audio recorded, transcribed, reviewed for accuracy, and entered into ATLAS.ti qualitative software (Version 8.4) for analysis (ATLASti.com, [5]). Two qualitative researchers (SGM and JJ) used a content analysis approach [14] to analyze emergent themes related to COVID-19’s impact on methadone treatment entry as well as continuing care in OTPs. Subcodes specific to the COVID-19-related methadone regulatory flexibilities were identified as either barriers or facilitators. The lead analysts then met periodically with the study PI (RPS) to discuss notes from the interviews, review coding, and develop code flow diagrams to contrast how these flexibilities impacted care for established patients already receiving methadone treatment and on people trying to enter methadone treatment during the pandemic.

## Results

States and OTPs made rapid changes to limit face-to-face contact and keep patients and staff as safe as possible during the COVID-19 pandemic while facilitating continued access to care. All the participating states adopted SAMHSA’s regulatory exemptions. While these exemptions were issued at the federal level, approval, coordination, and implementation were required at the state level. One SOTA described their role in aligning state guidelines with the federal exemptions in the following way:

*“And we had a number of opiate treatment programs reaching out to us because we had issued state-specific guidance that was in alignment with the federal government in allowing more flexibility to offer more services through things like telehealth, and then, also again to have more take-homes, so individuals wouldn’t have to come on-site physically for administration and dosing of medication.” (SOTA #1)*

This type of state-level guidance was both needed and welcomed by OTPs, who were trying to make rapid shifts in long-established operating procedures. However, according to the OTP staff and SOTAs interviewed for this study, as described below, the exemptions appear to have been more beneficial for established OTP patients than new program applicants.

### Impact on established patients

#### Take-home doses

Participants described methadone take-home flexibilities as essential in permitting treatment to continue while implementing the physical distancing recommendations to prevent COVID-19 transmission. Increased number of take-home doses resulted in fewer patients attending the clinic for methadone administration, which thereby reduced crowding.

*Well, now under [state office of addiction and support services] they’re saying those same people that they were limiting they have now made into a 3 day a week [methadone] pick-up, which means they’re only in the clinic Monday, Wednesday, and Friday. Or they’ve even given them once a week [methadone] pick-up where they only show up on a Monday. They dose in front of our nursing staff, they get six take-home bottles, and they come back the following Monday. (Intake Staff #1)*

The respondents indicated that the take-home flexibilities impacted patients and staff differently, with patients appreciating having to come to the clinic less often to receive their medication. In contrast, the OTP staff found that dispensing an increased number of take-home per patient required nurses to dispense up to 28 take-home methadone doses for some patients. This change created pressure on the nursing staff, as illustrated in the following quote by an OTP intake coordinator.

*So instead of the nurse pouring a single dose, watching them drink it, they pour the single dose, they watch them drink it, and then they have to sit there and pour twenty-eight take-home bottles, label twenty-eight take home bottles, make sure the clients take them with them and then move on to the next client. So, what would have normally taken five minutes at the window is now taking fifteen, twenty minutes and that’s another reason why we have to limit the amount of people in the building because otherwise they’ll be standing around all day. (Intake Staff #2)*

While a decreased number patients were in the facility each day, the extra take-homes changed the nurses’ workflow.

#### Telehealth for established patients

Participants reported that patients were able to benefit from remote counseling, which enabled them to continue receiving behavioral support without risking COVID-19 exposure from face-to-face visits for patients and staff.

*[SAMHSA] has allowed now, opiate treatment programs, very clearly in writing, to be able to use certain telehealth resources for both medical appointments, once the client has already been admitted to a treatment program, as well as counseling can be done now through telehealth. (SOTA #1)*

Much like with the changes in take-home flexibilities, the COVID telehealth changes created flexibility and alternative access to care during the need for physical distancing that otherwise would not have been possible during the pandemic.

*COVID is what really impacted our clinic, as far as like, with the counseling too. They weren’t receiving counseling and then we went to telehealth. (Leadership Staff # 1).*

Participants frequently mentioned the benefits of telehealth for increasing individual counseling participation among patients with particular barriers to clinic attendance, as illustrated by the following quote by an OTP director.

*I know eventually, once we’re able to manage the pandemic better, then I’m not sure, but I think that everybody that is involved with this system is looking at how people are benefited and how we’re able to provide more services and engage more individuals than in the past would engage in treatment because of having to come in, especially women with children when they used to bring their kids to the building, and now with COVID again we can’t allow that. So, I think it is actually providing a tool to continue to provide services so people can continue to engage. So yeah, absolutely our goal will be to continue to provide telehealth services. (Leadership Staff #2)*

This quote highlights how staff perceived that telehealth particularly benefitted patients who would have struggled to attend counseling during COVID, such as women with children. However, it appears that telehealth did not work for everyone.

Participants indicated that even established patients were not always able to participate in remote counseling due to lack of phone or computer access or privacy considerations at home. So, while counselors might have been working remotely, patients sometimes still needed to come into the clinics to access phones or computers to conduct their remote counseling sessions.

*It’s often a lot of chaos and crisis going on in their lives that needs to be dealt with their counselor, so it can be really, really difficult to reach them if they’re not on site. When we did tele-practice with my counselors, our population of people, whether it’s their cell phone numbers changing day to day, they don’t have a cell phone that supports the ability to do telemedicine. Actually, it was really, really difficult. And so, what we wound up doing was, even if my counselors were working remotely, we set up telehealth equipment in our extra rooms, in our extra offices on site and had the patient actually come in to do a session with the counselor via telehealth. So, we very quickly shifted to that because my counselors they were not getting in touch with people, people weren’t responding. (Leadership Staff #3)*

Telehealth was also consistently described as insufficient when it came to group counseling sessions. As illustrated in the following example provided by an intake staff member, group treatment largely ceased during COVID, despite the telehealth approval.

*Our group attendance was just abysmal. With the online we had a low adherence rate at that time, but I think, really, we were just trying to get through an unprecedented event as best we could and provide essential services basically. (Intake Staff #3)*

While individual sessions could more easily be accommodated via telehealth, group sessions, which typically comprise the majority of psychosocial support received in OTPs, were largely omitted from care during the pandemic.

### Impact on new program applicants

In contrast to the ways in which the regulatory flexibilities eased access to care for established patients, they did not appear to have the same favorable impact on program applicants.

#### Telehealth psychosocial assessments for new program applicants

Counseling and admission staff were often working remotely to limit COVID exposure, which hampered their ability to establish direct contact with people attempting to enter treatment, creating a barrier to admission. The staff noted that making contact simply to schedule remote intake appointments for eligibility screening or psychosocial assessments often proved difficult because phone numbers were not reliable. In addition, in some cases, people attempting to enter treatment were not willing or able to use telehealth.

*When everything switched to telehealth and we didn’t have people in the office for a little bit, we had some like the OAs, office assistants, and stuff, but most clinical staff wasn’t in and so we didn’t have the people making the connections with the people trying to get into treatment. Like we’d get an application and not know who the person was and then wouldn’t follow up and all that kind of stuff because of that kind of stuff. It really delayed getting people doctor’s appointments because some people couldn’t or wouldn’t do telehealth or didn’t know how and we couldn’t get the hands-on stuff that we needed usually to get people signed up. (Intake Staff #4)*

#### Histories and physicals

The regulatory flexibilities allowed individuals entering OTPs for buprenorphine treatment to be admitted following a remote telehealth medical assessment. However, as noted by one SOTA, SAMHSA did not permit remote medical assessments for admission to methadone treatment.

*You know there was like a little decline in new treatment for the first few months of the pandemic and then everything stabilized after that. So, at the OTPs, specifically, the relaxation was given at the federal level that if somebody wanted to get admitted on buprenorphine then they could do that through telehealth but that same relaxation wasn’t granted for people wanting to start methadone. (SOTA #2)*

Participants indicated that failing to permit telehealth medical assessments for methadone treatment admission made it difficult to limit the number of people in the clinic. Admissions were more challenging during the pandemic when medical personnel were in great demand and short supply. As the following quote exemplifies, physical distancing measures could not always be extended to the program applicants, who still needed to be on-site to complete the intake process.

*I guess that would be the biggest thing, but there’s still requirements for an in-person physical examination that has to occur for any new patient that comes in, and that still has to be done physically in-person with a prescriber… (SOTA #1)*

The pandemic also exacerbated the pre-COVID shortage of doctors and nurses, as medical staff became harder to recruit or retain during COVID because they sometimes needed to quarantine due to COVID infection or exposure.

*As it stands in any OTP, we operate on a strict medical staffing budget as well and we are granted exactly how many we need for our current numbers. And throughout COVID that has become more pronounced. We’re operating exactly to our max, as far as medical staffing, and then having one have to be out, for example, because they have to do quarantine for two weeks. (Leadership Staff #2)*

*…at the beginning of the pandemic we had one agency that ended up losing a few nurses to COVID early on in the pandemic, so it was one of these things that they were really having trouble. (SOTA #2)*

Prior to the pandemic, program applicants who could not be seen by a physician for admission, were either referred elsewhere, placed on a waitlist, or required to check in with the program repeatedly until a medical appointment could be made. This process was no different during COVID.

## Discussion

This study examined the views of OTP staff from six programs located on the east and west coasts of the USA and their SOTAs, on the impact of the COVID-19 pandemic and its associated federal and state COVID-19 Public Health Emergency (PHE) regulatory exemptions. These regulatory exemptions went into effect in March 2020, at the start of the parent study which examined the effectiveness of an implementation facilitation intervention to prompt the use of IM (methadone treatment without counseling) and other approaches to address admission delays. Under the COVID-19 PHE, SAMHSA allowed states to request a blanket exemption from the prior OTP take home regulations to permit the use of clinical judgment, rather than adherence to eight specific criteria and tenure in treatment requirements. When implemented, the PHE policies allowed OTPs to provide stable patients up to 28 take-home doses and to provide “less stable” patients (who were deemed able to responsibly handle their medication) with up to 14 take home doses (SAMHSA, [32]). The pandemic, with SAMHSA’s support, also permitted the use of remote telehealth counseling via video or telephone [11].

Although states were not required to implement the expansion of take-home doses, all of the SOTAs in the present study quickly did so. McIlveen and colleagues noted that 45 states (of the 49 with OTPs) requested the blanket exemption [24]. Prior to the PHE, 10 states prohibited take-home doses in the first 30 days of treatment and seven prohibited take-home doses during the first 90 days of treatment [34]. Going forward, states are reviewing their policies based on their experiences during the pandemic to ensure clinically appropriate and accessible care.

Participants reported that the response to the COVID-19 pandemic provided more benefit to established patients than program applicants. Established patients were able to obtain a greater number of take-home doses more quickly than prior to the pandemic to increase physical distancing at the programs. Several reports have noted the benefits of these regulatory flexibilities including enhanced patient-centered care, improved patient-provider rapport, increased patient autonomy and engagement [1, 13, 21, 33]. A mixed methods study found that take-home dose flexibliity among stable patients was associated with receiving more take homes, higher rates of treatment retention, and lower rates of opioid positive drug tests [13]. Patients in that study reported that the increased number of take homes supported their recovery because they felt trusted by the staff, spent less time traveling to the program, which permitted increased time spent on work and recreation, and were less exposed to drug-using patients at the program.

Several studies indicate that prior concerns about the impact of increases in take-homes resulting in methadone diversion-related problems and methadone overdose death do not appear to have been realized [3, 6, 10, 20, 35, 37, 38]. While OTPs were able to provide patients with the new maximum number of take-home doses, several studies found that OTP staff used their discretion in determining who should get additional take home doses [11, 18]. Going forward, the impact of expanded take-home availability should be monitored [20].

Hatch-Maillette and colleagues [12] raised a caution that the greater flexibility in granting take-home doses under the PHE could potentially negatively impact patient equity given the lack of specificity in the definitions of what constituted a “stable” or “less stable” patient. SAMHSA issued proposed permanent regulatory revisions in December 2022 [9] and updated temporary regulatory guidance in April 2023 to extend take-home flexibility for 1 year while the final regulatory revisions go in effect.

Both the guidance and the proposed final regulations have only six (rather than eight in the pre-pandemic regulations) specific take-home criteria. These criteria are more specific than those of “stable” and “less stable” under the original PHE declaration but are much less prescriptive than those prior to the PHE. This middle ground should mitigate any concern of potential bias in approving take-homes. The guidance and proposed regulations also considerably reduce the tenure in treatment requirement, eliminate the reliance on complete abstinence of any drug, and leave take-home eligibility up to the clinical judgment of the provider [9]. These specific criteria should be helpful to both providers and patients to safely expand access to care.

Participants reported that the expansion of take-home dose availability required adjustments to the clinic workflow. Although nurses were administering methadone to fewer patients each day, each patient took longer because of the need to prepare and dispense a greater number of take-home doses. The newly-proposed SAMHSA revisions to the OTP regulations may help address this issue because they expand the definition of qualified practitioner who can administer or dispense methadone [9]. SAMHSA noted that this proposed change will permit greater staff flexibility within states that permit a wider variety of disciplines to administer and/or dispense methadone.

Established patients also benefited from the expanded use of remote telehealth counseling. This too was quickly approved by the participating SOTAs. OTP staff participants reported that attendance at counseling had dropped precipitously at the start of the pandemic prior to the use of telehealth. OTPs adjusted their treatment approach to permit patients to attend individual counseling sessions remotely (typically by telephone), although challenges remained for patients with limited resources. Such sessions were often limited to telephone voice counseling rather than by video because patients lacked access to smartphones with data plans or computers with cameras, microphones and internet connections.

Participants’ reports of attendance at individual counseling attendance during the pandemic once remote services were offered did not extend to group counseling, which was largely put on hold during the present study. Greater problems in implementing group counseling during the pandemic was found by other OTPs [11]. Since group counseling in OTPs prior to the pandemic was predominant, the shift to individual telehealth counseling was notable, though its impact on outcomes is not known. The drop in group counseling attendance was consistent with findings from a nine-state patient survey [27]. Another survey of 100 addiction treatment providers in California found that they believed remote individual counseling was as effective as in-person individual counseling, but they were less sure about the relative effectiveness of telehealth-delivered group counseling [23].

The impact of the switch from group to individual counseling is not known because of limited effectiveness data in OTPs on in-person group vs. individual counseling and limited effectiveness data on remote counseling [22]. We are aware of only one randomized trial of remote vs. in-person individual counseling in an OTP which found that both conditions had similar attendance and rates of positive drug tests [17]. Similarly, a small, randomized trial from the same group comparing remote vs. in person group counseling found no significant difference in attendance or in achieving two consecutive weeks of drug abstinence [17].

It should be noted that a randomized trial that compared interim methadone treatment without counseling to methadone treatment with counseling found no significant differences in treatment retention or illicit opioid use during the first 4 months of treatment [29]. More research is needed on the effectiveness of remote counseling and comparing individual to group counseling. Programs should weigh the benefits of increasing patient access to telehealth to support patient autonomy and equity and to remove the burden of commuting to the program while juggling responsibilities of work, childcare, and criminal justice supervision [12]. As noted in the present study and by others [23] this would be particularly beneficial to patients with care giving responsibilities.

In the present study, staff reported that program applicants did not benefit as much as established patients from the COVID-19 PHE flexibility because the increases in take-home doses and remote counseling were not relevant to the admission process. SAMHSA did permit medical assessments for OTP admission for buprenorphine treatment to be conducted remotely, however this was not permitted for methadone treatment. Thus, permitting other remote intake activities, including eligibility screening and psychosocial assessments, did not help program applicants gain admission more rapidly. Participants identified that medical staff shortages as a bottleneck to admissions prior to COVID were exacerbated during the pandemic because medical providers had to quarantine when they or someone in their household had been exposed to COVID. The medical staff shortage coupled with the in-person requirement for physical exams created strain on the OTPs in our study.

During the first year of the COVID pandemic, given the known association between retention in methadone treatment and the reduced risk of opioid overdose death [19, 30], implementing clinically appropriate options that aid access to services was critical. Post pandemic, the proposed SAMHSA revisions to the OTP regulations would permanently permit admission medical assessments for new patients via video (not voice only), or as an alternative, to permit the use of a physical exam conducted within seven 7 days of admission by a non-OTP. These flexibilities should help to facilitate new admissions. They would also permit the current temporary take-home flexibilities and expansion of qualified providers to remain in effect permanently.

This study has several limitations. First, it was conducted as part of an implementation facilitation study of interim methadone treatment and involved OTPs with treatment entry delays that preceded COVID-19. Thus, it may not generalize to clinics that were not experiencing treatment delays prior to COVID. Second, only six clinics in four states on the east and west coasts and their respective SOTAs participated in the study. Therefore, findings may not generalize to OTPs in other states. Third, the focus of the parent study was not on COVID regulatory flexibilities at the outset, so findings emerged in response to other questions about clinic processes related to the parent study. A study that focused specifically on COVID-related changes may have uncovered additional areas that were not mentioned due to the lack of topical overlap. Finally, the first wave of interviews referred to treatment activities occurring prior to the broad availability of COVID vaccinations in the US and may not reflect OTP treatment occurring today.

## Conclusions

The present findings can inform SAMHSA’s proposed OTP regulatory reform which includes, among other important changes, expanding access to take-home doses and to telehealth, permitting a greater variety of staff to administer/dispense methadone in keeping with state regulations, and permitting provider medical assessment for admission to be conducted via TeleVideo or by a provider outside of the OTP. Should future pandemics or other disasters require easing of particular aspects of the OTP regulations, findings from OTP staff and SOTAs indicate that specific regulatory exemptions can be effective in increasing access to services.

## Data Availability

The datasets used and analyzed during the current study are available from the corresponding author on reasonable request.

## Abbreviations

OTPs: Opioid treatment programs
SUD: Substance use disorder
SAMHSA: Substance Abuse Mental Health Services Administration
DEA: Drug enforcement agency
SSAs: Single state agencies
COVID-19: Coronavirus disease
NIDA: National Institute on Drug Abuse
SOTA: State Opioid Treatment Authorities
WIRB: Western Institutional Review Board
